# Platelet GPIIb supports initial pulmonary retention but inhibits subsequent proliferation of melanoma cells during hematogenic metastasis

**DOI:** 10.1371/journal.pone.0172788

**Published:** 2017-03-02

**Authors:** Katrin Echtler, Ildiko Konrad, Michael Lorenz, Simon Schneider, Sebastian Hofmaier, Florian Plenagl, Konstantin Stark, Thomas Czermak, Anca Tirniceriu, Martin Eichhorn, Axel Walch, Georg Enders, Steffen Massberg, Christian Schulz

**Affiliations:** 1 Medizinische Klinik und Poliklinik I, Klinikum der Universität, Ludwig-Maximilians-Universität, Munich, Germany; 2 Walter-Brendel-Zentrum für Experimentelle Medizin, Ludwig-Maximilians-Universität, Munich, Germany; 3 Medizinische Klinik, Klinikum rechts der Isar, Technische Universität München, Munich, Germany; 4 Chirurgische Klinik, Klinikum der Universität, Ludwig-Maximilians-Universität, Munich, Germany; 5 Research Unit Analytical Pathology, Helmholtz Zentrum München, German Research Center for Environmental Health, Neuherberg, Germany; Monash University, AUSTRALIA

## Abstract

Platelets modulate the process of cancer metastasis. However, current knowledge on the direct interaction of platelets and tumor cells is mostly based on findings obtained *in vitro*. We addressed the role of the platelet fibrinogen receptor glycoprotein IIb (integrin αIIb) for experimental melanoma metastasis *in vivo*. Highly metastatic B16-D5 melanoma cells were injected intravenously into GPIIb-deficient (GPIIb^-/-^) or wildtype (WT) mice. Acute accumulation of tumor cells in the pulmonary vasculature was assessed in real-time by confocal videofluorescence microscopy. Arrest of tumor cells was dramatically reduced in GPIIb^-/-^ mice as compared to WT. Importantly, we found that mainly multicellular aggregates accumulated in the pulmonary circulation of WT, instead B16-D5 aggregates were significantly smaller in GPIIb^-/-^ mice. While pulmonary arrest of melanoma was clearly dependent on GPIIb in this early phase of metastasis, we also addressed tumor progression 10 days after injection. Inversely, and unexpectedly, we found that melanoma metastasis was now increased in GPIIb^-/-^ mice. In contrast, GPIIb did not regulate local melanoma proliferation in a subcutaneous tumor model. Our data suggest that the platelet fibrinogen receptor has a differential role in the modulation of hematogenic melanoma metastasis. While platelets clearly support early steps in pulmonary metastasis via GPIIb-dependent formation of platelet-tumor-aggregates, at a later stage its absence is associated with an accelerated development of melanoma metastases.

## Introduction

Hematogenic tumor cell dissemination is the leading cause of death in patients with malignant melanomas. The lung is among the most common sites of melanoma metastasis [1], while extrapulmonary metastases usually do not occur prior to lung metastasis [2]. Melanoma patients with lung metastases have a 5-year survival rate of below 10% [3]. Hence, strategies targeting hematogenic melanoma cell spreading are considered promising in order to improve the overall outcome of melanoma patients [4]. Hematogenic tumor cell dissemination engages a multi-step process. Once released from the primary tumor, metastatic cells invade the vasculature and travel in the blood stream to reach their target organs. Tumor cell dissemination is a highly inefficient process as more than 99% of tumor cells are rapidly eliminated in the circulation [5]. In order to survive, circulating tumor cells have to be retained within organs. Upon arrest, they transmigrate into the perivascular matrix, where they proliferate and may ultimately lead to the clinical manifestation of cancer metastasis [6].

Several studies have linked platelets to hematogenic tumor cell dissemination in various malignancies [7, 8](reviewed in [9]), however, the exact role of platelets has remained controversial. It has been shown that platelets directly interact with certain tumor cells under both static and low flow conditions [7, 8, 10, 11]. In addition, studies mostly performed *in vitro* suggest that platelets promote tumor cell arrest on subendothelial matrices [8, 12], and foster tumor cell proliferation [13]. Platelets are also essential for regulating the hemostasis of tumor vasculature and for preventing intratumoral hemorrhage [14]. Recently, platelets have been demonstrated to impair natural killer (NK) cell-mediated elimination of tumor cells by binding to tumor cell surfaces [15, 16]. Although these findings suggest a supportive role for platelets in tumor cell spreading and growth, the exact contribution and biological relevance of platelets for metastasis is still unclear. While some studies have shown that targeting of platelet membrane receptors, such as glycoprotein (GP)IIb-IIIa, by monoclonal antibodies or elimination of circulating platelets results in a significant reduction in the number of metastases in transplantable murine tumor models [11, 17], others report that inhibition of platelet receptors does not confer protection against tumor cell dissemination or rather increases metastasis formation. In fact, inhibition of platelet GPIbα was demonstrated to enhance hematogenic cancer metastasis [18, 19]. While the number of pulmonary metastases was the major endpoint in most of the above studies, they differ substantially with respect to the timing and duration of inhibition of platelet membrane receptors. Based on the discrepant findings in different experimental settings, platelet receptors possibly have opposing effects on the different steps of initial tumor cell dissemination and subsequent tumor cell proliferation during metastasis formation. However, their role for distinct steps in the process of hematogenic tumor cell metastasis *in vivo* has not been addressed in detail to date. In addition, transgenic mice deficient in GPIIb-IIIa, which resemble the phenotype of human Glanzmann thrombasthenia [20], have not been studied in this context, and determining hematogenic metastasis in such mice seems of broad interest [21].

In the present study, we dissected the role of platelet αIIb integrin (GPIIb) for early and late steps in pulmonary melanoma metastasis. We first addressed *in vitro* potential mechanisms for initial recruitment of circulating melanoma cells to vascular endothelium using a flow chamber model and then assessed the role of GPIIb for metastasis formation *in vivo* in mice lacking integrin αIIb (GPIIb^-/-^) [20]. GPIIb associates with GPIIIa (integrin β3) to form the platelet-specific integrin heterodimer GPIIb-IIIa (integrin αIIbβ3), representing the most abundant platelet surface receptor and predominantly functioning as platelet fibrinogen receptor. By binding to fibrinogen, but also to von Willebrand factor, GPIIb-IIIa mediates cross-linking of adjacent platelets, resulting in platelet aggregation and platelet secretion of chemokines as well as growth factors [22, 23]. Moreover, binding of GPIIb-IIIa to fibronectin, vitronectin or PECAM-1 leads to platelet adhesion to the vessel wall [24]. In order to follow the initial steps of tumor metastasis in wildtype (WT) and GPIIb-deficient mice, we applied a novel microscopic approach using a fluorescence optical imaging system based on laser scanning confocal technology. We show that the acute retention of malignant melanoma cells is dramatically reduced in mice deficient in platelet GPIIb. We also found that GPIIb has a minor effect of adhesion of single melanoma cells, but rather mediates the formation of platelet-rich melanoma cell aggregates, which are retained in the pulmonary vasculature. Despite defective initial tumor cell accumulation, mice lacking GPIIb were not protected from pulmonary metastasis formation, but rather revealed a significant increase in metastatic tumor growth and proliferation in the lung 10 days after melanoma injection. Together, this provides evidence that platelet GPIIb contributes to initial tumor cell arrest at the early stage of tumor cell dissemination, but prevents subsequent metastatic tumor growth and/or survival.

## Material and methods

### Animals

All mice were on C57BL/6J background. GPIIb^-/-^ mice (αIIb-integrin)-deficient mice were generated as described previously [20]. Age- and sex-matched GPIIb^+/+^ (WT) littermates served as controls. Animals were housed in specific pathogen free conditions in individually ventilated type III cages from TECNIPLAST (Hohenpeißenberg, Germany). Mice received standard chow from Altromin (Lage, Germany) and sterile tap water ad libitum. Appropriate enrichment (plastic-houses from TECNIPLAST, sterile pulp paper and coarsely litter) was provided. Physical condition of mice was monitored twice daily. A protocol approved by the Government of Bavaria for early euthanasia of potentially ill or moribund mice was in place. All experimental procedures on animals met the requirements of the German legislation on protection of animals and were approved by the Government of Bavaria, Germany.

### Antibodies

For evaluation of melanoma surface molecule expression by flow cytometry, PE-conjugated rat anti-mouse αv-monoclonal antibody (mAb) (clone RMV-7), anti-β3-mAb (clone HM beta3.1) and rat IgG_2a_-isotype control were obtained from BD Pharmingen (Heidelberg, Germany). anti-αIIb-mAb (clone MWReg 30) was obtained from eBioscience. FITC-conjugated mouse anti-human αvβ3-mAb (clone LM609) was purchased from Chemicon, mouse anti-human αIIb-mAb (clone P2) from Immunotec (Beckam Coulter, Krefeld, Germany) and IgG isotype control (X 0927, X 0928) from Dako (Glostrup, Denmark). For evaluation of platelet surface molecule expression by flow cytometry, anti-β3-mAb (BD Biosciences, clone 2C9.G2), anti-αIIb-mAb (eBioscience, clone MWReg30), GPIb (Emfret Analytics, clone X488), GPVI (Emfret Analytics, clone JAQ1), GPIX (Emfret Analytics, clone Xia.B4), anti-P-Selectin antibody (BD Biosciences, clone RB40.34), Annexin V (BD Pharmingen) and respective isotype controls were used. For fluorescence microscopy, polyclonal FITC-conjugated anti-GFP and goat rhodamine-conjugated anti-PE secondary antibody from Rockland (Gilbertsville, PA, USA), anti-ki67-PE (clone B56) from BD Biosciences (Cat. No. 556027, Germany), rabbit polyclonal anti-fluorescein/Oregon Green IgG (Molecular Probes, Karlsbad, CA, USA), rat anti-mouse αIIb-mAb (clone MWReg 30, BD Pharmingen), and anti-rat Alexa Fluor 488 and Alexa Fluor 594 (Molecular Probes) were purchased. For flow chamber experiments, c7E3 Fab (ReoPro) was obtained from Lilly (Bad Homburg, Germany).

### Evaluation of platelet surface molecule expression

Expression of platelet surface molecules was investigated in washed murine platelets. Whole blood was obtained from GPIIb^+/+^ and GPIIb^-/-^ mice by puncture of the inferior vena cava and anticoagulated with sodium citrate. Whole blood was washed and centrifuged in the presence of 0.5 μg prostaglandin. Washed platelets were resuspended in Tyrode’s buffer. Resting washed platelets were incubated with antibodies indicated above and surface expression was measured by flow cytometry. Surface P-Selectin- and AnnexinV/PS-exposure was determined in resting and Thrombin (0.05 U/mL, 10 min) stimulated platelets.

### Cell culture

B16-D5 mouse melanoma cells, a poorly immunogenic subclone of the spontaneously arising B16-BL6 melanoma, were kindly provided by Dr. Winter (Franz Cancer Research Center, Earle A. Chiles Research Institute, Portland, OR) [25, 26]. αvβ3-positive M21 and αvβ3-negative M21L human melanoma cells were kindly provided by Prof. Dr. Haubner (Dep. of Nuclear Medicine, Medical University of Innsbruck, Austria) [27]. Cryo-preserved pooled primary human umbilical vein endothelial cells (HUVEC) were obtained from Promocell (Heidelberg, Germany) and cultured as described [28]. Cells were grown until confluence, and either left untreated (resting) or stimulated with 50 ng/mL TNF-α and 20 ng/mL IFN-γ for 20 hours before flow chamber experiments were performed.

### Intravital microscopy

Mice were anesthetized by intraperitoneal application of Midazolam (10mg/kg body weight, Ratiopharm, Ulm, Germany), Medetomidin (1mg/kg, Pfizer, Karlsruhe, Germany) and Fentanyl (0,2mg/kg, Curamed Pharma, Munich, Germany). A polyethylene catheter was introduced into the left jugular vein. Upon tracheotomy mice were intubated and mechanically ventilated (MiniVent Typ 845, Hugo Sachs Electronics, March, Germany) at 120 strokes/minute and 150μl stroke volume of 0,5% isofluran-oxygen ratio [29]. Subsequently, a partial right thoracotomy with costal resection was performed to exhibit an oval lung field of approximately 1,5 x 2 cm^2^. To minimize movements of the diaphragma, which readily conduct to the lung, the right phrenical nerve was anesthetized by local application of 1% Lidocaine (B. Braun, Melsungen, Germany). B16-D5 cells were labeled with 5-carboxyfluorescein diacetate succinimidyl ester (DCF) and adjusted to 1x10^5^ cells in 250μl Tyrodes buffer. DCF-tagged B16-D5 were intravenously infused in syngeneic mice on C57BL/6J background. For experiments conducted *in vivo*, GPIIb^-/-^ mice and their sex-matched GPIIb^+/+^ littermates were handled as treatment pairs, thus receiving cells from the same preparation. In another set of experiments, 1.5x10^8^ isolated WT or GPIIb^-/-^ platelets were administered into GPIIb^-/-^ mice prior to B16-D5 application where indicated. Immediately and 1 hour after B16-D5 application the B16-D5 arrest in the pulmonary vasculature was monitored *in vivo*. Using confocal laser scanning fibre bundle microscopy [30] (Cellvizio^TM^, Mauna Kea Technologies, Paris, France) we directly visualized the DCF-tagged tumor cells *in vivo* (S3 Fig). A flexible fibre bundle microprobe (MiniO Proflex microprobe, diameter 300μm, Mauna Kea) with a maximal optical penetration depth of 150μm was placed on the lung surface. The vasculature of the dorsal, ventral and basal pulmonary margin of each mouse was visualized in identical chronological order and direction. All images were taken during a ventilation pause following exhalation. Videos were recorded in real-time at a speed of 12 frames per second for subsequent analysis. To quantify melanoma retention in the lung, the number of retained tumor cell clusters was counted in the total range of the dorsal, ventral and basal right pulmonary margin from the recorded videos. To evaluate the size of accumulated tumor cell aggregates in the pulmonary vasculature, the first 10 events imaged at the dorsal margin were selected and their area was subsequently assessed using computer-assisted image analysis software (CapImage, Dr. Zeintl, Heidelberg, Germany).

### Evaluation of B16-D5 metastasis formation

To determine the effect of GPIIb-IIIa mediated platelet action on metastasis formation in the lung, 1x10^5^ B16-D5 were infused into the jugular vein of GPIIb^+/+^ (WT) or GPIIb^-/-^ mice. As described above, GPIIb^-/-^ mice and their sex-matched GPIIb^+/+^ littermates were studied as treatment pairs. 10 days later, animals were euthanized by cervical dislocation and the number of metastatic nodules on the surface of the right lung was counted by blinded staff, and thereafter processed for histological analysis as described below. In addition, mRNA was isolated from the left lung of the same animal and pre-melanosomal protein (Pmel; Mus musculus 16756-Si: Silver) expression was quantified using Real-Time PCR (Quantitec Primer Assay, QT00102487, Qiagen, Hildesheim, Germany). Total mRNA was isolated with the RNeasy Midi Kit (Qiagen) following manufacturer’s instructions, and further reverse transcribed into cDNA using the High Capacity cDNA Reverse Transcription Kit (Applied Biosystems, Darmstadt, Germany). Quantitative Real-Time PCR was performed with SYBR Green (Fermentas, Leon-Rot, Germany) as a fluorescent dsDNA-binding dye and Pmel expression was quantified by the ΔCt-Method after normalization to a housekeeping gene (beta-actin). All reactions were run as triplicates.

### Histology

1) Histological analysis of GFP^+^ B16-D5 interaction with platelets: Lung specimens were obtained after injection of 0.5x10^6^ GFP^+^ B16 and incubated overnight at 4°C in a fixative containing 0.01M sodium periodate, 0.075M L-lysine and 1% paraformaldehyde. Thereafter, tissues were washed with 0.1M phosphate buffer, passed through sucrose gradient solutions and embedded in OCT compound (TissueTek®, Sakura, Netherlands). Cryostat sections (7 μm) were stained with the following reagents: 4',6-Diamidino-2-phenylindol (DAPI), rat anti-mouse GPIIb (CD41) and an Alexa Fluor 594-conjugated anti-rat antibody. To enhance the GFP-signal of B16-D5 we used a FITC-conjugated anti-GFP antibody (Rockland, Rockland, PA). Stained sections were mounted in Prolong Gold anti-fade mounting medium (Thermo Fisher Scientific, Waltham, MA).

2) Ki67 staining of lung specimen. After determining colonization, lungs were fixed in 10% neutral buffered formalin solution, dehydrated and embedded in paraffin. 20 sections of 3 μm thickness with 50 μm distance were generated from each lung. The sections were rehydrated and heat induced antigen retrieval was achieved by cooking the slides in citrate buffer (pH 6). Fc-blockade (BD Bioscience) was performed for 30 minutes. For sequential double immunofluorescence analysis sections were first treated with anti-ki67 PE (BD Biosciences) and a goat rhodamine-conjugated anti-PE secondary ab (Rockland), and then with an anti-HMB-45 mAb (Agilent, Santa Clara, CA) and a goat anti-mouse Alexa-488 secondary ab (Invitrogen™, Thermo Fisher Scientific), and DAPI. For detection of melanoma cells within the pulmonary tissue we applied the mouse anti-human antibody HMB-45 raised against the melanoma-specific marker Pmel. Human and mouse Pmel17/gp100 proteins have 79.7% amino acid sequence homology [31]. The mAb HMB-45 thus recognizes a shared epitope between both species, and murine B16 melanoma cell lines expressing Pmel17 demonstrated consistent staining with HMB-45 [32, 33]. Sections were investigated using a Leica DMRB epifluorescence microscopy.

### Role of GPIIb for tumor growth

Wild-type and GPIIb-deficient mice were anesthetized and their back was shaved. 10^5^ B16-D5 cells in 200ul PBS were injected subcutaneously into the skin of the right flank. From day 14 after subcutaneous implantation, tumor diameters were measured every other day using a caliper. The tumor was explanted on day 21 post-injection and its weight was measured. The majority of mice from both genotypes developed a visible tumor within few days. Three mice of each genotype did not develop significant tumors during the whole duration of the experiment.

### Assessment of platelet-tumor interactions under flow conditions

1) Interaction of human platelets with M21 melanoma cells. Whole blood was drawn from healthy donors and platelets were isolated as described [28]. All blood donors were volunteers who gave their free and informed written consent to this research study, which conforms to the ethical standards of the Declaration of Helsinki. Approval was obtained from the Technical University of Munich institutional review board for these studies. Flow chamber experiments were basically performed as reported earlier [28]. In brief, HUVEC were cultivated to confluency on glass cover slips for usage in a flow chamber, and either left untreated or co-stimulated with 50 ng/mL TNF-α and 20 ng/mL IFN-γ for 20 hours. HUVEC were perfused with DCF-labeled M21 or M21L at a shear rate of 1000/s for 10 minutes in the presence or absence of platelet-rich plasma or washed human platelets. To block GPIIb-IIIa receptor specific action, melanoma cells or the platelet-melanoma cell suspensions were preincubated with the monoclonal antibody abciximab (c7E3 Fab, 4 μg/mL; Lilly Deutschland, Bad Homburg, Germany) for 15 minutes. Tumor-endothelium interaction and tumor aggregate formation was studied in a flow chamber system mounted on the stage of an Axiovert 100 (Zeiss, Jena, Germany) microscope. All images were videotaped and subsequently evaluated offline using a computer-assisted image analysis program (Cap Image 7.1, Dr. Zeintl, Heidelberg, Germany). The number of firmly adherent melanoma cells was assessed by counting the cells that did not move or detach from the endothelial monolayer within 20 seconds. Further, the number of tumor cells within platelet-melanoma-aggregates was quantified.

2) Interactions of murine platelets with B16 melanoma cells. Whole blood was drawn from mice by cardiac puncture and platelets were isolated as described [34]. 10^6^ B16 cells were perfused together with 10^8^ washed murine platelets for 20min at 37C. Calcium (1mM), magnesium (1mM) and fibrinogen (Sigma, 75 μg/ml) were supplemented [35]. Perfusion was performed at 1000/s in a closed flow chamber system allowing recirculation of cells at continuous unidirectional and pulsatile flow (Ibidi, Martinsried, Germany). Platelet tumor aggregate formation was determined by flow cytometric analysis of platelet (glycoprotein Ib, CD42) positive cells (as compared to PE-isotype control) within the B16-population. Data was quantified on a FACS Calibur(Becton Dickinson, Franklin Lakes, NJ) and evaluated using FlowJo v10, LLCsoftware (Ashland, OR)

### Determination of platelet-tumor cell aggregate formation *in vitro*

M21 melanoma cells were incubated for 15 minutes in the absence and presence of platelet-rich plasma and subsequently analyzed by fluorescence and electron microscopy. For fluorescence microscopy, probes were stained with fluorescent-labeled phalloidin and fluorescent antibodies directed against CD41 and fibrinogen. For electron microscopy (EM), cells were fixed in a sodium-cacodylate buffer (pH 7.4) with glutaraldehyde (2.5%) and tannin (0.02%). For scanning EM (SEM) fixed cells were plated on coverslips, and for transmission EM (TEM) probes were embedded in epoxyresin. Electron microscopy was performed as previously described [36].

### Statistics

Prism 5.0 (GraphPad, San Diego, CA) was used as statistical software. Data are presented as means ± SEM unless stated otherwise. For *in vivo* experiments with matched treatment pairs data were analyzed using Student’s paired two-sample t-test. Other data was analyzed using the Tukey-test or one-way analysis of variance (ANOVA) with a Tukey posthoc test where adequate. A value of P < 0.05 was regarded as significant.

## Results

### Platelet-melanoma interaction under flow conditions

**Fig 1:**
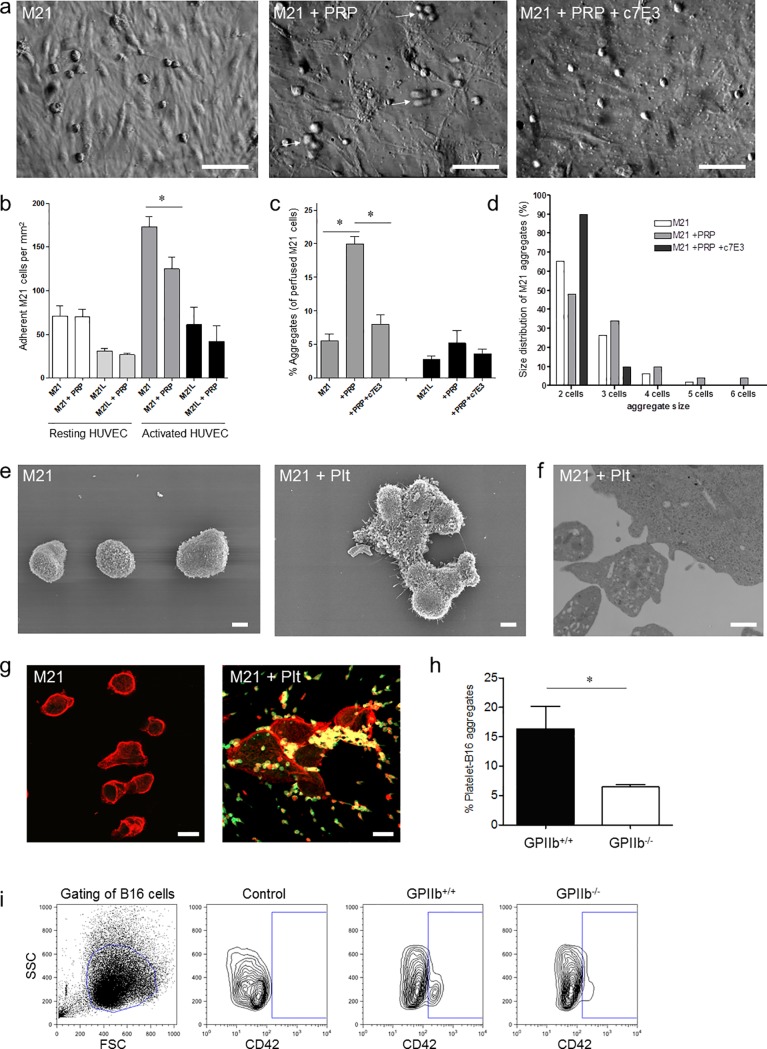
Platelet-tumor-endothelium interaction under flow. **a)** Photomicrographs show platelet and M21 aggregate formation under flow (1000/s). Co-perfusion of platelets and M21 cells induced formation of aggregates which did not firmly adhere and are therefore only partly focused (middle picture; arrows indicate platelet-M21 aggregates). Aggregation is virtually abolished in the absence of platelets (left picture) or the presence of c7E3 Fab (right picture). Bars 0.2mm. Images were taken using a Zeiss Axiovert 100 microscope, 20x objective. **b)** HUVEC (resting or TNF-α/IFN-γ-stimulated) were perfused with M21 or αv-deficient M21L melanoma cells, that lack the expression of both αvβ3 and αvβ5 integrins, in the absence and presence of platelet-rich plasma (10min, shear rate 1000/s). Results are shown as number of firmly adherent M21 and M21L (n = 5; *P<0.05 for M21 vs. M21+PRP on activated HUVEC). **c)** Frequency of M21 and M21L aggregates in the absence and presence of platelets and c7E3 antibody. Results are given as number of melanoma cell-aggregates in % of all perfused tumor cells (n = 5; *P<0.001 for M21 in the absence vs. presence of platelets). **d)** Size distribution of M21 aggregates in the absence and presence of platelets and c7E3. **e-g)** M21 melanoma cells were incubated for 15 minutes in the absence and presence of platelet-rich plasma. **e)** Scanning electron microscopy indicates single melanoma cells in the absence (left picture) and melanoma cell aggregates in the presence of platelets (right picture). Bars, 5μm. **f)** Transmission electron microscopy indicates direct platelet-melanoma interaction. Bar, 1μm. **g)** Photomicrographs show representative fluorescence microscopy images of M21-platelet aggregates stained with phalloidin (red) and anti-GPIIb (CD41) mAb (green). Bars, 20μm. Images were taken using a Leica DMRB epifluoresence microscope, 20x objective. **h-i)** Analysis of mouse GPIIb^-/-^ or WT platelet interaction with B16 melanoma under flow conditions (1000/s). **h)** Percentage of platelet-positive B16 cells (n = 4; *P<0.05). **i)** Representative FACS contour plots indicate CD42-staining on B16 cells in the absence (control) and presence of GPIIb^+/+^ or GPIIb^-/-^ platelets.

Adhesion of tumor cells in the target vasculature is the initial step of metastatic seeding. Activated platelets have recently been shown to promote tumor cell adhesion to endothelial cells *in vitro* under venous flow conditions [8, 10]. However, in the *in vivo* setting, tumor cells are exposed to higher shear forces and are likely to encounter mostly non-activated platelets. Therefore, we first addressed whether unstimulated platelets–similar to collagen-activated platelets [8]–are also able to promote tumor cell adhesion, when exposed to capillary flow *in vitro*. Yet, when we applied shear conditions resembling those prevailing in the pulmonary microcirculation [37], only few M21 human melanoma cells, and even less M21L melanoma cells, lacking the major adhesion receptor αvβ3 [27], adhered to resting endothelial cells under flow (Fig 1A, left image and Fig 1B). Addition of non pre-activated platelet-rich plasma did not increase the number of adherent melanoma cells (Fig 1A, middle image and Fig 1B). Only when we co-stimulated endothelial cells with 50ng/mL TNF-α and 20ng/mL IFN-γ for 20 hours adhesion of melanoma cells was significantly increased (Fig 1B). Yet again the presence of platelets did not significantly increase, but rather reduced the adhesion of tumor cells to the endothelial monolayer (Fig 1B). Together, this suggests that non pre-activated platelets are dispensable for adhesion of single melanoma cells to endothelial cells under capillary flow conditions.

### Formation of platelet-rich tumor cell aggregates *in vitro*

Previous *in vitro* data have identified that platelets bind to melanoma cells in a process involving platelet GPIIb-IIIa, fibrinogen and tumor cell-expressed αvβ3 integrin [8]. We hypothesized that rather than promoting tumor cell adhesion, GPIIb-IIIa mediates the formation of platelet–melanoma cell aggregates. In a first step, we analyzed *in vitro* whether platelet–melanoma cell aggregate formation occurs under flow. We perfused M21 and M21L in the presence or absence of platelets over a confluent endothelial monolayer applying capillary flow conditions. We found that in the absence of platelets, melanoma cells circulated as single cells, while aggregates of M21 cells (~6% of perfused melanoma cells), or αvβ3-deficient M21L cells were barely observed (<3% of perfused melanoma cells) (Fig 1A, left image, 1C and 1D). In contrast, in the presence of platelets, aggregate formation of M21 was significantly increased; approximately 20% of all melanoma cells were now incorporated into aggregates (Fig 1A, middle image and Fig 1C and 1D). When we examined the size of aggregates, we found that the few aggregates which formed in the absence of platelets mostly consisted of two cells, whereas larger aggregates were rarely detected (Fig 1D). In contrast, when platelets were added to/co-perfused with M21 cells, the aggregate size significantly increased; in fact, the number of aggregates containing 3–4 cells was approximately 5-fold higher in the presence of platelets, when compared to M21 cells in the absence of platelets. We even frequently found large aggregates comprising >5 cells in the presence of platelets (Fig 1D). Further analysis of the melanoma cell aggregates by immunofluorescence and electron microscopy revealed that platelets physically participated in melanoma cell aggregation. We observed numerous platelets/platelet aggregates interspersed between the assembled melanoma cells (Fig 1E–1G). The heterotypic platelet-melanoma cell aggregation under flow required GPIIb-IIIa. Correspondingly, aggregate formation was strongly reduced in the presence of the antibody c7E3 Fab targeting both GPIIb-IIIa and the vitronectin receptor αvβ3 integrin (Fig 1A, right image, Fig 1C and 1D).

We added another set of experiments to determine whether in murine cells GPIIb integrin also plays a role in platelet-tumor aggregate formation under capillary flow conditions. To do so, we isolated platelets from GPIIb^+/+^ and GPIIb^-/-^ mice and co-perfused them with murine B16-D5 melanoma cells in the presence of fibrinogen in a closed flow chamber system [35]. Consecutive quantification by flow cytometry analysis revealed that also in the murine system platelet-B16 aggregate formation was enhanced in the presence of GPIIb (Fig 1H and 1I). To test whether GPIIb-deficiency alters platelet surface receptor expression, flow cytometry was performed with isolated GPIIb^+/+^ and GPIIb^-/-^ platelets. We found no difference in surface expression of glycoprotein GPIb-IX-V complex or GPVI receptor in GPIIb^-/-^ platelets compared to GPIIb^+/+^ controls. Further no difference in P-Selectin or Annexin V expression could be detected upon thrombin activation (S2A–S2C Fig). Taken together, these findings implicate that under *in vitro* capillary flow conditions non pre-activated platelets do not promote adhesion of single melanoma cells to endothelial cells, but rather trigger the formation of platelet-tumor cell aggregates which is in part dependent on GPIIb-IIIa integrin. Flow chamber assays, however, are not capable of resembling the complex *in vivo* capillary flow conditions, such as alternating shear rates, tube diameters or variable site densities of endothelial adhesion molecules which all may influence melanoma cell adhesion and aggregation behavior [38, 39]. Hence, we designed mouse experiments to define whether platelet-melanoma cell aggregate formation also occurs *in vivo* and whether this mechanism is of any relevance for pulmonary metastasis.

### Circulating platelet-melanoma aggregates form *in vivo* and are retained in the pulmonary vasculature

**Fig 2:**
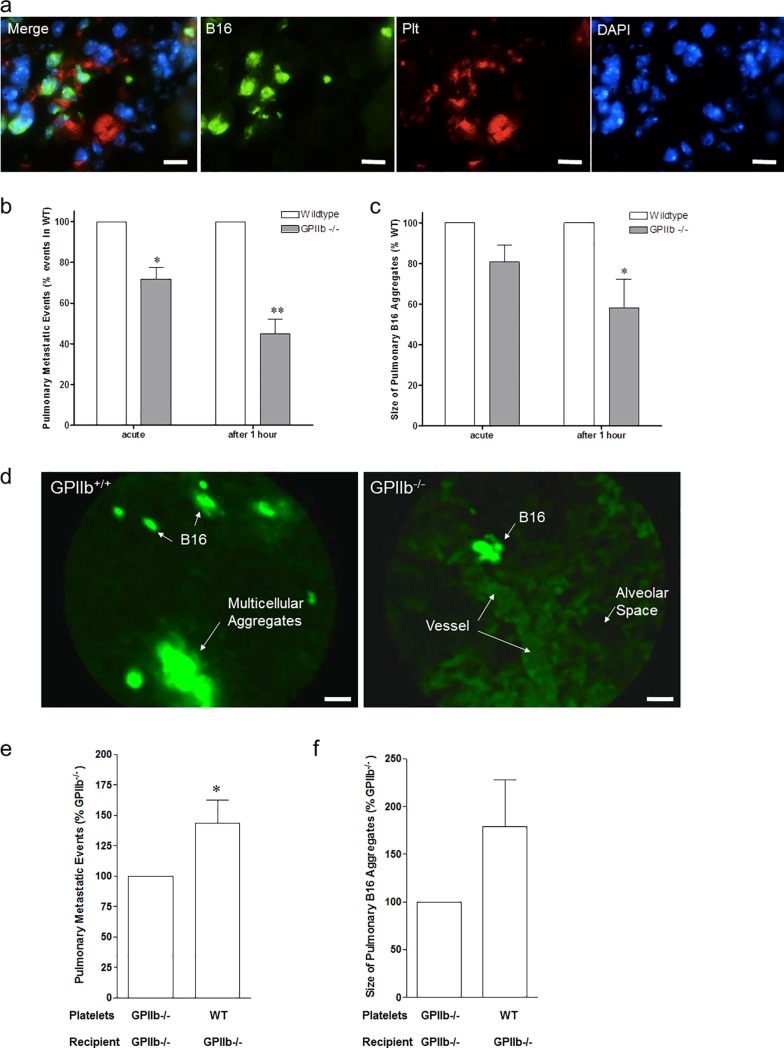
Platelet-tumor-aggregate formation *in vivo*. **a)** GFP-transfected B16-D5 melanoma cells were injected intravenously into wildtype mice. After 1 hour, lung tissue was obtained for immunofluorescence analysis. Photomicrographs show mouse lung tissue stained with antibodies directed against platelet GPIIb (CD41, red) and B16-D5 (GFP, green); nuclei were stained with DAPI (blue). Bars, 40μm. Images were taken using a Leica DMRB epifluoresence microscope, 20x objective. **b-d)** Arrest of DCF-tagged B16-D5 melanoma cells was visualized in the pulmonary vasculature by intravital confocal videofluorescence microscopy (IVM) in GPIIb^+/+^ and GPIIb^-/-^ littermate mice immediately and after 1 hour. **b)** Number of metastatic events was quantified. Results are given as percentage of firmly adherent B16-D5 in GPIIb^-/-^ mice compared to its WT littermate (n = 5–6 experiments per group; *P<0.01 acute; **P<0.001 after 1 hour). **c)** Size of B16-aggregates was quantified. Results are given as percentage of B16-aggregate size in GPIIb^-/-^ mice compared to its WT littermate (n = 5–6; *P<0.05 after 1 hour). **d)** Photomicrographs show representative IVM images obtained in GPIIb^+/+^ and GPIIb^-/-^. In GPIIb^+/+^ mice, arrest of large multicellular aggregates is frequently observed (left). Arrest of DCF-tagged B16-D5 is visualized in precapillary vessels (right). Bars, 20μm. **d e-f)** GPIIb^+/+^ (WT) or GPIIb^-/-^ platelets were injected into GPIIb^-/-^ mice just prior to administration of DCF-labeled B16-D5. IVM was performed immediately and after 1 hour. **e)** Number of metastatic events was quantified. Results are given as percentage of firmly adherent B16-D5 in GPIIb^-/-^ littermates receiving GPIIb^-/-^ platelets (n = 4; *P<0.05). **f)** Size of B16-aggregates was quantified. Results are given as percentage of B16-aggregate size in GPIIb^-/-^ littermates receiving GPIIb^-/-^ platelets (n = 3; P = n.s.).

To evaluate intravascular conglomeration of melanoma cells, we infused 0.5 x 10^6^ GFP-expressing B16-D5 into the jugular vein of anaesthetized, spontaneously breathing WT mice. One hour after B16 application, citrated blood was carefully drawn from the right ventricle and lungs were harvested. Using flow cytometry, we determined numerous CD41-positive platelets attached to GFP^+^ B16-D5 in mouse whole blood (figure not shown), suggesting that platelet-induced melanoma-aggregate formation occurs under flow both *in vitro* and *in vivo*. When we examined the lungs of these animals one hour after infusion of GFP^+^ B16-D5 melanoma cells, we found large aggregates consisting of B16-D5 cells and platelets within the lung capillary network (Fig 2A). Thus, platelet-dependent aggregate formation in the blood and lungs is an early event after tumor cell infusion into mice *in vivo*.

### Determination of melanoma cell-aggregate embolization in the pulmonary microcirculation using IVM

Based on the above findings we hypothesized that rather than adhesion of individual tumor cells, the platelet-dependent formation of melanoma cell-aggregates and their subsequent retention within the pulmonary circulation triggers efficient metastatic melanoma cell seeding. In order to address this hypothesis, we analyzed *in vivo* whether blood-borne tumor cell-platelet aggregates can indeed be trapped within the pulmonary microcirculation. We therefore directly visualized the recruitment of melanoma cells in the pulmonary vasculature using real-time *in vivo* confocal microscopy (IVM) of the mouse pulmonary vasculature (S3 Fig). DCF-labeled B16-D5 cells were administered into the jugular vein of anaesthetized and mechanically ventilated WT and GPIIb^-/-^ mice. Immediately after cell infusion, numerous melanoma cells accumulated in the WT lung vasculature. IVM revealed that B16-D5 melanoma cells are rarely recruited to the WT pulmonary circulation as single cells, but mostly accumulate in the form of multicellular aggregates captured in larger vessel (S1 Videos). In several occasions B16-D5 were entrapped at vascular bifurcations. In contrast, significantly less melanoma cells accumulated in pulmonary vessels of GPIIb^-/-^ mice within the first minutes after infusion (Fig 2B). Moreover, when we quantified the size of B16-D5 conglomerates, we found that there was not only a decrease in the overall number of retained aggregates but also that B16-D5 aggregates were significantly smaller in GPIIb^-/-^ mice compared to WT animals (Fig 2B and 2C). The difference between WT and GPIIb^-/-^ mice was even more pronounced 1 hour after B16-D5 administration (Fig 2B and 2C). This suggests that a substantial proportion of those melanoma cells initially retained in GPIIb^-/-^ mice was able to pass the pulmonary microcirculation after prolonged transit times.

GPIIb-IIIa has been considered to be expressed exclusively on cells derived from the megakaryocytic/platelet lineage. However, recent evidence suggests that also some tumor cell lines surface express GPIIb-IIIa [40, 41]. Nevertheless, when we performed flow cytometry we found that neither B16-D5 nor M21 melanoma cells expressed GPIIb (S1 Fig). In order to exclude that GPIIb-IIIa from additional (non-platelet, non-melanoma cell) sources contributes to melanoma cell-aggregate formation and retention *in vivo*, we performed an additional set of experiments in which GPIIb^-/-^ mice were treated with GPIIb^-/-^ or WT platelets prior to administration of DCF-labeled B16-D5. After 1 hour, IVM of the pulmonary vasculature was performed as described above. When WT platelets were infused into GPIIb^-/-^ mice we now observed an increase in number of tumor cells that accumulated in the pulmonary vasculature compared to GPIIb^-/-^ mice receiving GPIIb^-/-^ platelets (Fig 2E and 2F). This indicates that melanoma cell recruitment in the pulmonary microcirculation is mediated by platelets and depends on platelet GPIIb-IIIa. At the same time this set of experiments makes it unlikely that in GPIIb^-/-^ mice a pulmonary phenotype restricts tumor cell accumulation.

Taken together, our *in vivo* imaging revealed that platelet-melanoma cell coaggregation rather than adhesion of single melanoma cells is essential for their initial capturing in the pulmonary circulation. This mechanical entrapment of large platelet-tumor cell aggregates could—at least in part—explain the pulmonary tropism of malignant melanoma.

### Role of platelet GPIIb for melanoma metastasis after 10 days

**Fig 3:**
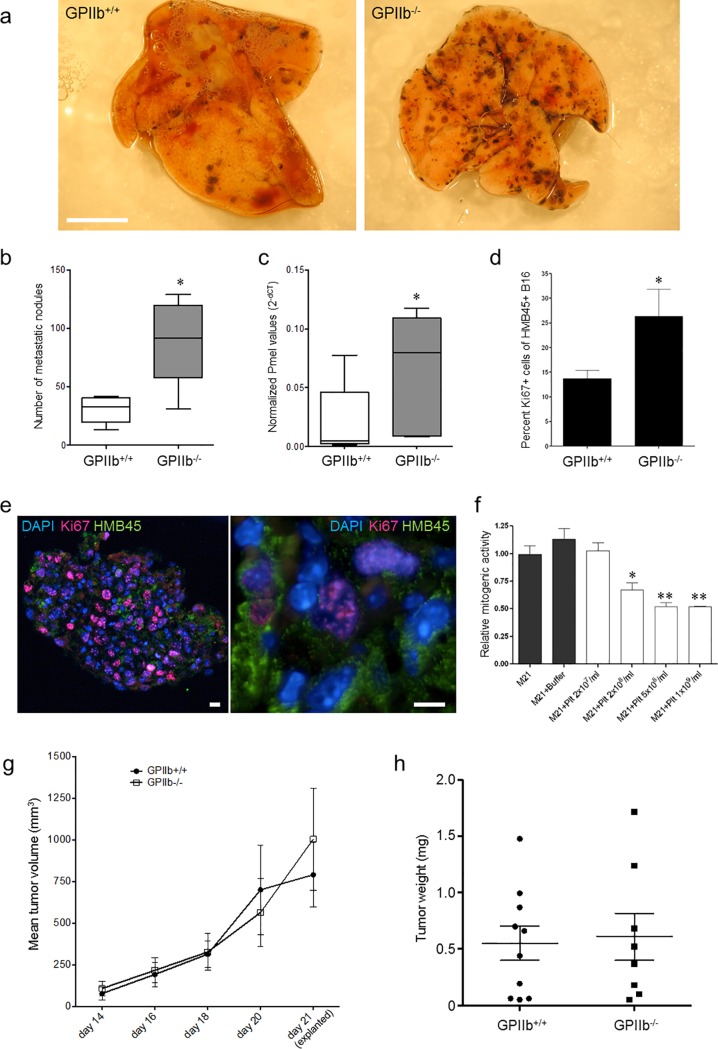
Effect of platelet GPIIb on melanoma metastasis formation. **a)-c)** B16-D5 melanoma cells were injected intravenously into GPIIb^+/+^ or GPIIb^-/-^ littermate mice. After 10 days, metastasis formation was analyzed. **a)** Photomicrographs show representative lung images. Bars, 0.5 cm. **b)** Quantification of metastatic nodules in the lung (n = 5; *P<0.05). Boxes indicate median, whiskers indicate min and max values. **c)** Quantification of Pmel mRNA expression. Individual Pmel data normalized to beta-actin is indicated as 2^(-ΔCt)^ (n = 5; *P<0.05). Boxes indicate median, whiskers indicate min and max values. **d-e)** Pulmonary melanoma proliferation was determined by analyzing Ki67 expression in HMB-45-positive melanoma cells 10 days after intravenous seeding (n = 5; *P<0.05). Photomicrographs show fluorescence microscopy images of mouse lung tissue stained with antibodies directed against melanoma HMB-45 (green) and Ki67 (red); nuclei were stained with DAPI (blue). Images show 3μm optical sections. Left image, overview of lung tissue section; right image, magnification. Bars, 100μm. Images were taken using a Leica DMRB epifluoresence microscope, 20x and 40x objective. **f)** Effect of platelets on M21 mitogenesis *in vitro*. Serum-starved M21 cells were grown in the absence or presence of increasing concentrations of washed human platelet and BrdU uptake was quantified (*P<0.05, **P<0.01). **g-h)** Effect of platelet GPIIb on subcutaneous melanoma growth. **g)** B16-D5 cells were injected subcutaneously in the right flank of wildtype and GPIIb^-/-^ littermate mice and tumor growth surveyed using a digital scaliper. **h)** 21 days after tumor cell seeding tissues were explanted. Tumor weight was measured indicating no difference in subcutaneous tumor growth (P = n.s.).

We next aimed to further dissect the relevance of platelet GPIIb-mediated events in hematogenic melanoma cell metastasis beyond the acute effects on tumor cell arrest. To do so, we intravenously infused B16-D5 cells into WT and GPIIb^-/-^ mice. Ten days later the right lungs were harvested and the numbers of metastases counted in situ. Unexpectedly, we observed that despite the defect in initial melanoma cell retention in GPIIb^-/-^ mice, the mutants were not protected from metastasis formation at later stages. In fact, the number of metastases was significantly lower in lungs of GPIIb^+/+^ littermates compared to GPIIb^-/-^mutants (Fig 3A–3C).

In order to further evaluate the tumor load ten days after B16-D5 application, we analyzed the expression of melanoma cell specific antigen Pmel17/silver in the lungs. In line with macroscopic findings, mRNA expression of Pmel17/silver revealed an attenuated tumor load in GPIIb^+/+^ mice as compared to their GPIIb^-/-^ littermates (Fig 3C).

Taken together, these findings have three major implications: (1) a GPIIb-IIIa mediated platelet mechanism is essential to allow initial tumor cell retention in the pulmonary vasculature; (2) despite favoring initial melanoma cell retention, GPIIb-IIIa-mediated platelet activity suppresses subsequent tumor growth at later stages after initial seeding; (3) consistent with recent findings in the brain microcirculation [6] our present observations in the lung indicate that initial seeding and micrometastasis formation are not inevitably linked to efficient growth to macrometastases.

### Reduced melanoma proliferation in the presence of platelets and GPIIb

To address this apparent discrepancy and to further evaluate our observation that a platelet GPIIb-dependent mechanism inhibits melanoma growth after initial seeding, we checked for melanoma cell proliferation rates 10 days after melanoma cell injection using Ki67 staining on lung sections. For identification of melanoma cells within the pulmonary tissue we used the antibody HMB-45 raised against the melanoma-specific marker Pmel. We found that in GPIIb^-/-^ mice significantly more melanoma cells were Ki67 positive compared to their GPIIb^+/+^ littermates (Fig 3D and 3E, S4 Fig). This indicates that the presence of GPIIb suppresses tumor cell proliferation in our model of melanoma metastasis formation.

To address whether platelets can directly influence melanoma growth, we next performed cell culture experiments of melanoma cells in the presence or absence of washed platelets. We studied incorporation of the thymidine analog bromodeoxyuridine (BrdU) as a marker for DNA synthesis and cell proliferation. We found that M21 melanoma proliferation was significantly reduced by addition of isolated human platelets. Notably, the effect was dose-dependent as incubation with increasing platelet numbers resulted in greater inhibition of tumor growth (Fig 3F). Thus, platelets, which accumulated in high numbers around melanoma cells *in vitro* (Fig 1A–1I) and *in vivo* (Fig 2A), increased initial tumor cell arrest and metastasis formation *in vivo* (Fig 2B–2D), but negatively regulated tumor cell proliferation (Fig 3D–3F).

### GPIIb does not impact on extravascular tumor growth

The experiments described above focused on hematogenic metastasis formation. To further assess the role of GPIIb in tumor growth, B16-D5 cells were injected subcutaneously in the dorsal skin of WT and GPIIb-deficient mice. Tumor size in both groups increased over time and the growth curves largely overlapped (Fig 3G). After 21 days, explant tumor weight was not significantly different between GPIIb^+/+^ (0.551 ± 0.14 g) and GPIIb^-/-^ (0.608 ± 0.21 g) (Fig 3H). These results demonstrate that the effect of GPIIb was restricted to formation for hematogenic metastases.

## Discussion

Platelet involvement in hematogenic cancer metastasis has been recognized for several years, however their exact role in this process remains controversial [21]. While some studies have indicated that inhibition of platelet adhesion receptors or platelet depletion provides protection against metastatic tumor dissemination [17], others reported that targeted inhibition of certain platelet surface receptors may even result in enhanced experimental tumor metastasis [18]. Current data imply that the role of platelets in tumor metastasis is more complex than initially anticipated. In particular, the impact of platelets on distinct, sequential steps in the cascade of tumor metastasis have not been elucidated, in part because until recently metastatic organ sites such as the lung were not accessible to direct *in vivo* examination.

In the present study we used intravital confocal fiber bundle microscopy to directly visualize initial tumor cell seeding in a mouse melanoma model of hematogenic metastasis. We found that melanoma cells were recruited to the lungs in form of tumor cells aggregates, which had been cross-linked by blood platelets in a process involving GPIIb-IIIa. This platelet-mediated coaggregation of blood-borne tumor cells significantly increased their ability to home to the pulmonary microcirculation. While platelets in this way promoted initial micrometastasis, they attenuated subsequent tumor cell proliferation and growth to macrometastases. Correspondingly, mice with defective tumor-platelet aggregation due to genetic ablation of GPIIb-IIIa integrin revealed attenuated initial micromatastasis but enlarged pulmonary melanoma macrometastases ten days after tumor cell infusion.

Previously, it was assumed that tumor cells accumulate in target tissues primarily as single cells that directly adhere to the vascular wall. Indeed earlier evidence suggests that melanoma cells have the capacity to firmly adhere to components of the subendothelial matrix under dynamic flow conditions [42]. However, during dissemination in the pulmonary microcirculation tumor cells most likely encounter an intact pulmonary endothelial monolayer, rather than exposed extracellular matrix proteins. *In vitro*, melanoma cell lines are also able to adhere to endothelial cells in a process involving αvβ3 integrin [42, 43], α4β1-integrin, endothelial VCAM-1, Lu-ECAM-1, as well as the chemokines, including CXCL12 [44, 45]. However, so far melanoma cell-endothelial cell interactions were tested mostly under low venous flow conditions, which predominate in microcapillaries and postcapillary venules [37]. Consistent with previous reports [6], we observed *in vivo* that melanoma cells are recruited mostly to precapillary arterioles, rather than accumulating in postcapillary venules (authors unpublished observation). Yet, when we exposed melanoma cells to shear conditions typical for precapillary vascular segments, only few M21 cells managed to adhere to an endothelial monolayer. Hence, adhesion of individual melanoma cells to the endothelium is unlikely to contribute substantially to melanoma cell seeding in the lungs. Instead our data indicate that the entrapment of tumor cells aggregates tied up by platelets in a GPIIb-IIIa-dependent manner is the primary mode of metastatic seeding.

GPIIb-IIIa, the platelet fibrinogen receptor, was required for the formation of melanoma cell-platelet aggregates. Correspondingly, loss or inhibition of platelet GPIIb-IIIa abolished tumor cell-platelet interaction. Although GPIIb-IIIa expression was reported in some malignant cell lines [40, 41], we excluded any potential contribution from non-platelet expressed GPIIb-IIIa: (1) neither M21 nor B16-D5 melanoma cells used in this study express GPIIb-IIIa and (2) the *in vivo* defect in melanoma cell aggregation and pulmonary retention in GPIIb^-/-^ mice could be rescued by infusion of wildtype platelets. Further we excluded that GPIIb^-/-^ platelets revealed alterations in GPIb-IX-V or GPVI adhesion receptor expression or indicated impaired platelet activation (S2A–S2C Fig). Although previous studies addressed a potential contribution of platelet GPIIb-IIIa for hematogenic tumor cell dissemination, these studies were limited in that they used antibodies or GPIIIa-deficient transgenic mice in order to define the role of GPIIb-IIIa [46]. While the GPIIb-IIIa receptor complex is platelet-specific, the GPIIIa (ß3 integrin) subunit also associates with another α subunit to form the vitronectin receptor (αvß3 integrin), expressed by endothelial cells, osteoclasts, and other cells including some tumor cells (reviewed in [47]). In contrast GPIIb (αIIb integrin) does not associate with other subunits than GPIIIa. Furthermore, antibodies such as 10E5 and 7E3 or the F(ab’)_2_ variant of 7E3, bind to both GPIIb and GPIIIa on cells [48]. Hence, our present study is the first to specifically demonstrate a role for platelet GPIIb-IIIa in the context of hematogenic melanoma metastasis. Consistent with previous reports [8], our present findings suggest that platelet GPIIb-IIIa integrin associates with αvβ3-integrin, abundantly expressed by M21 and B16-D5 melanoma cells. Accordingly, depletion of GPIIb, function-blocking antibodies to GPIIb-IIIa integrin or depletion of αvβ3-integrin in M21L virtually abolished the formation of platelet-tumor cell interactions under flow conditions.

Once attached to circulating tumor cells in a GPIIb-dependent manner, platelets not only support arrest by formation of platelet-melanoma-aggregates which are retained in the pulmonary vasculature. Platelets also establish a microenvironment that may modulate tumor cell proliferation and angiogenesis [15, 49]. Although defective platelet-tumor cell interaction due to loss of GPIIb impaired initial tumor cell retention, it led to a marked increase in tumor burden 10 days after hematogenic pulmonary metastasis. This is an unexpected finding as previous reports using blocking antibodies directed against GPIIb-IIIa in rodents reported an inhibitory effect on tumor growth [18, 50]. However, with off-target modulation of integrins other than platelet GPIIb-IIIa, antibody treatment may not reveal the exact role of platelet GPIIb-IIIa in cancer metastasis. In fact, our present findings are consistent with the recent observation that the absence of the platelet receptor GPIbα leads to enhanced cancer metastasis 10 days after melanoma injection [18]. A similar observation was made in mice deficient in von Willebrand factor, a multimeric glycoprotein that mediates blood coagulation and platelet adhesion via GPIbα and GPIIb-IIIa [51]. While in the latter studies the underlying mechanisms remained elusive, we report here that platelets critically inhibit melanoma growth. In lung tissue sections, analysis of Ki67 expression revealed a reduction in melanoma cell proliferation in animals with wildtype GPIIb-IIIa compared to GPIIb deficient littermates. However, this observation was clearly dependent on the pathological setting as subcutaneous tumor growth was not affected by the absence of GPIIb-IIIa.

In conclusion, we identify GPIIb-IIIa—an important platelet receptor—to differentially affect initial and subsequent steps of experimental pulmonary metastasis. Visualizing pulmonary micrometastasis *in vivo*, we demonstrate that platelet-specific GPIIb mediates the formation of platelet-tumor cell aggregates, which are captured in the pulmonary (micro-) circulation. While GPIIb in this way supports initial steps in pulmonary metastasis, GPIIb-mediated platelet-melanoma cell colocalization hampers subsequent tumor cell proliferation and impedes the development of melanoma macrometastases. Together, platelet fibrinogen receptor GPIIb-IIIa modulates distinct, sequential steps in the cascade of hematogenic metastasis with oppositional effects. The data elucidate numerous conflicting reports on the role of platelet function in melanoma metastasis and support our understanding of platelet-driven effects in melanoma biology.

## Supporting information

S1 Fig Surface expression analysis of melanoma cells. Mouse B16-D5 and M21 melanoma cells were assessed by flow cytometry for their surface expression of the integrins αIIb (GPIIb, CD41), αv (CD51), β3 (CD61) or IgG isotype control (rat IgG2b for mouse B16-D5, and mouse IgG1 for human M21) (n = 2–6, mean + SD).
(PDF)

S2 Fig Receptor expression and activation of murine platelets. Washed platelets preparations were generated from GPIIb^+/+^ and GPIIb^-/-^ mice and analysed by flow cytometry (n = 4). A) Surface expression of the integrins αIIb (GPIIb, CD41) and β_3_ (CD61), the glycoproteins Ib, IX and VI, and IgG isotype control stainings were measured. B-C) Comparison of P-selectin (CD62P) (B) and Annexin V (C) surface expression after Thrombin (0.1 U/mL) stimulation.
(PDF)

S3 Fig Schematic for intravital imaging. Mice were anesthetized and intravenously infused with DCF-tagged B16-D5. Using confocal laser scanning fibre bundle microscopy we directly visualized the DCF-tagged tumor cells *in vivo*. A flexible fibre bundle microprobe with a maximal optical penetration depth of 150μm was placed on the lung surface. The vasculature of the dorsal, ventral and basal pulmonary margin of each mouse was visualized as indicated in the schematic.
(PDF)

S4 Fig Control staining of lung tissue sections. Immunohistochemistry with isotype control antibodies for HMB45 and Ki67. Nuclei were stained with DAPI (blue). Images were taken with 20-fold magnifications. Images were taken using a Leica DMRB epifluoresence microscope, 20x objective.
(PDF)

S1 Videos Intravital microscopy of melanoma cells in the pulmonary vasculature.DCF-tagged B16-D5 melanoma cells were visualized in the pulmonary vasculature by intravital confocal videofluorescence microscopy (IVM) in GPIIb^+/+^ and GPIIb^-/-^ littermate mice after 1 hour. Images were taken using a Cellvizio confocal laser scanning fibre bundle microscope with a MiniO Proflex microprobe. Optical penetration depth was 150μm. a) Imaging of GPIIb^+/+^ (WT) mouse, b) GPIIb^-/-^ littermate.
(ZIP)
