# Supplementary figures and images for: Platelet GPIIb supports initial pulmonary retention but inhibits subsequent proliferation of melanoma cells during hematogenic metastasis
Source: PLoS One. 2017 Mar 2;12(3):e0172788. doi: 10.1371/journal.pone.0172788 (PMC5333841; doi:10.1371/journal.pone.0172788)

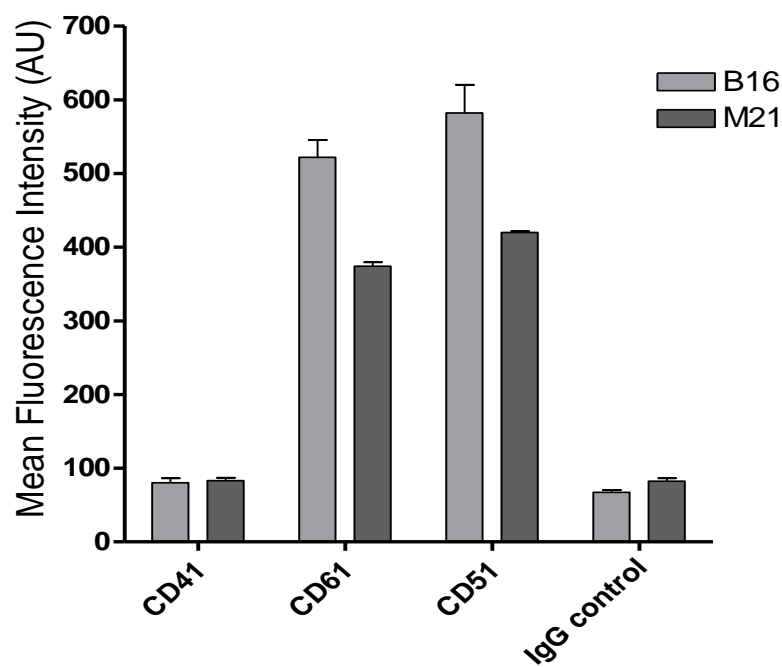

Supplement: S1 Fig — Mouse B16-D5 and M21 melanoma cells were assessed by flow cytometry for their surface expression of the integrins αIIb (GPIIb, CD41), αv (CD51), β3 (CD61) or IgG isotype control (rat IgG2b for mouse B16-D5, and mouse IgG1 for human M21) (n = 2–6, mean + SD). (PDF) [file pone.0172788.s001.pdf]

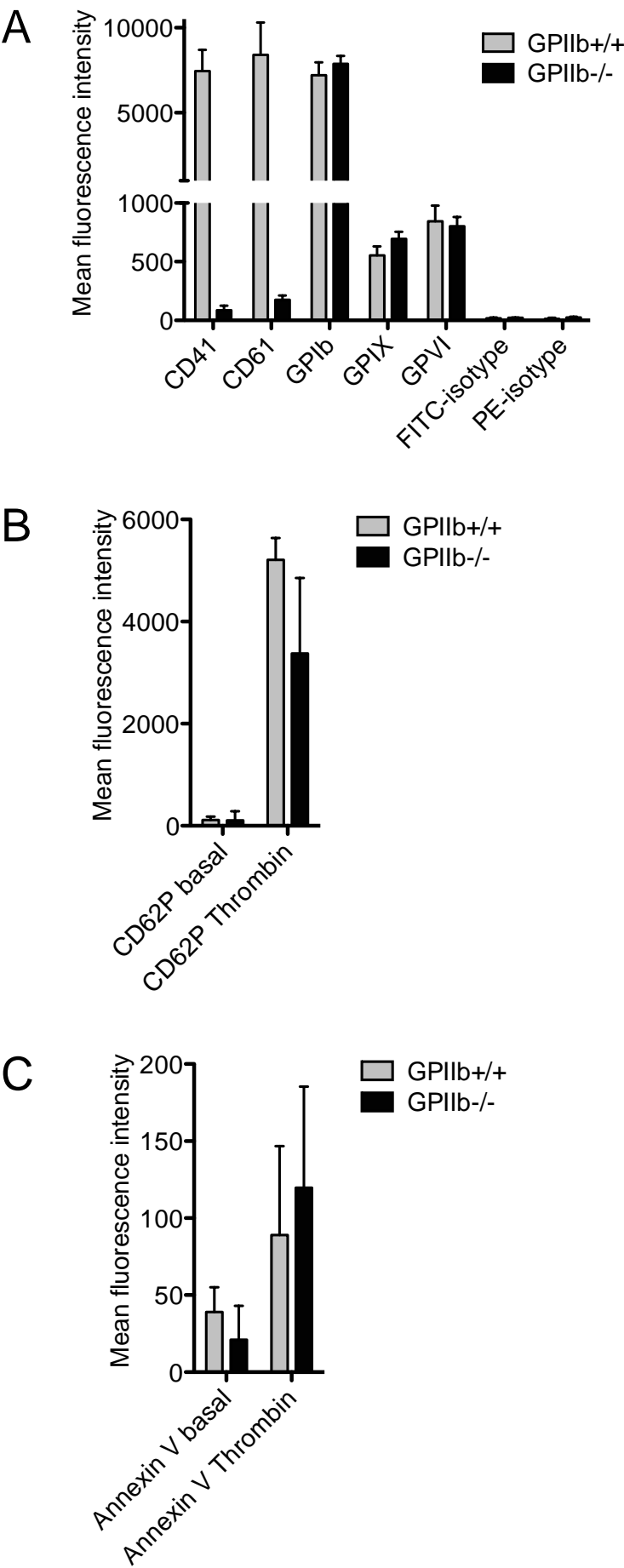

Supplement: S2 Fig — Washed platelets preparations were generated from GPIIb+/+ and GPIIb-/- mice and analysed by flow cytometry (n = 4). A) Surface expression of the integrins αIIb (GPIIb, CD41) and β3 (CD61), the glycoproteins Ib, IX and VI, and IgG isotype control stainings were measured. B-C) Comparison of P-selectin (CD62P) (B) and Annexin V (C) surface expression after Thrombin (0.1 U/mL) stimulation. (PDF) [file pone.0172788.s002.pdf]

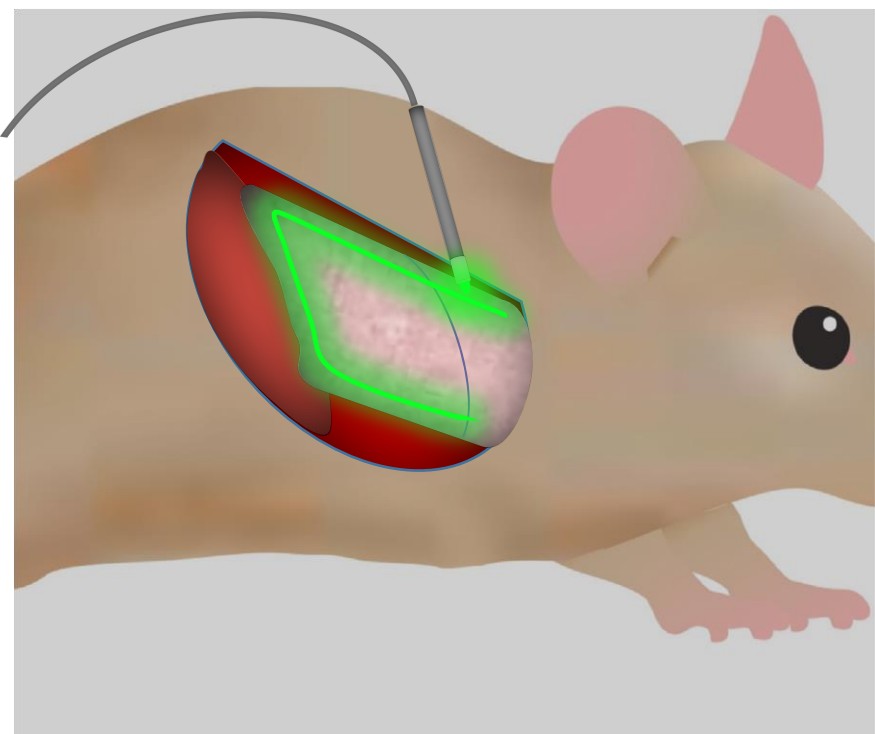

Supplement: S3 Fig — Mice were anesthetized and intravenously infused with DCF-tagged B16-D5. Using confocal laser scanning fibre bundle microscopy we directly visualized the DCF-tagged tumor cells in vivo. A flexible fibre bundle microprobe with a maximal optical penetration depth of 150μm was placed on the lung surface. The vasculature of the dorsal, ventral and basal pulmonary margin of each mouse was visualized as indicated in the schematic. (PDF) [file pone.0172788.s003.pdf]

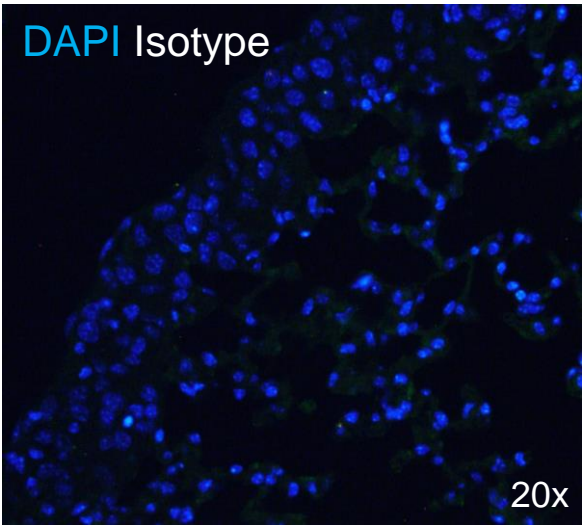

Supplement: S4 Fig — Immunohistochemistry with isotype control antibodies for HMB45 and Ki67. Nuclei were stained with DAPI (blue). Images were taken with 20-fold magnifications. Images were taken using a Leica DMRB epifluoresence microscope, 20x objective. (PDF) [file pone.0172788.s004.pdf]
